# The Latin American Call for Young Researcher Editorials: Identifying the Public Health Challenges in One of the Most Unequal Regions in the World

**DOI:** 10.3389/ijph.2023.1606830

**Published:** 2023-12-08

**Authors:** Germán Guerra, Ana Cecilia Quiroga Gutiérrez, Diana Buitrago-Garcia, Jonila Gabrani, Peter Francis Raguindin

**Affiliations:** ^1^ Institute of Global Health, Faculty of Medicine, University of Geneva, Geneva, Switzerland; ^2^ Global Health Program, Centre for Health Systems Research, National Institute of Public Health, Cuernavaca, Mexico; ^3^ Institute of Health Economics and Health Policy, Bern University of Applied Sciences, Bern, Switzerland; ^4^ Faculty of Health Sciences and Medicine, University of Lucerne, Lucerne, Switzerland; ^5^ Institute of Social and Preventive Medicine, University of Bern, Bern, Switzerland; ^6^ Faculty of Medicine, University of Medicine Tirana, Tirana, Albania; ^7^ Faculty of Medicine and Health Sciences, University of Ghent, Ghent, Belgium; ^8^ Institute of Child Health and Human Development, National Institutes of Health, University of the Philippines Manila, Manila, Philippines

**Keywords:** Latin America, inequalities of health, challenges, global health, research

**The IJPH series “Young Researcher Editorial” is a training project of the Swiss School of Public Health.**

Despite significant social and economic development in recent decades, Latin America remains one of the most unequal regions in the world [1]. According to estimates from United Nations Development Programme (UNDP), income inequality in Latin America is much higher than expected for its development level [2]. In 2022, while 10% of the top richest households possessed 77% of the regional wealth, the bottom poor 50% shared just 1% [3]. Moreover, one out of three persons in Latin America live in poverty and are at risk of experiencing the dire health consequences of social inequality [4].

With this in mind, we, the editors of the IJPH series “Young Researcher Editorial” (YRE) opened a call for YRE entitled “*Public Health in Latin America: Challenges and Perspectives*” in April 2022. The purpose of this call was to learn from what young Latin American researchers perceive as relevant public health issues in one of the most unequal regions in the world. Moreover, this call was aligned with the IJPH’s decades-long tradition of supporting an editorial strategy focused on social inequalities in public health. These inequalities have been previously addressed in articles from the Latin American region [5–7].

We approached seven public health research institutions in all Latin America and enthusiastically waited for submissions from our colleagues. We received 33 manuscripts in total -an overwhelming response we did not expect and a clear statement that our peers in the region well used the opportunity to raise their pen in an YRE. Unfortunately, due to the limited number of places assigned for publishing editorials for this call, we regret having declined many excellent submissions.

Among all 33 submissions, the most frequent topics were: healthcare systems (14); health inequality and vulnerable groups (4); gender (3); human health resources (2); mental health (2) and other [(8, including COVID-19 (4); nutrition (1); conceptual debates (2); planetary health (1)]. After careful scrutiny and a rigorous review process, we accepted six editorials for publication [8] three from Brazil, one from Colombia, one from Ecuador, and one from Mexico. These interesting pieces address some of the most pressing public health concerns in Latin America from the perspective of the next-generation of public health professionals. The subjects of the editorials can be grouped into three broad themes.

Firstly, gender, as a driver for social equity, was one of the most pervasive issues in public health in Latin-America. The YRE by Cortés-Gallego ([9]; Colombia), and Gomes do Nacimiento and Alves de Andrade-Valença ([10]; Brazil), underscored the urgency of strengthening the mechanisms through which gender-based health inequalities can be tackled. The assurance of access to women’s education for claiming their rights enshrined in legislation, or the deconstruction of hegemonic masculinity under which treatments for men with epilepsy are built, are some instances to achieve gender equity and health.

Another topic was the health service improvements in Latin America, particularly for the underserved populations in confined environments or with unmet healthcare needs. Yañez ([11]; Ecuador), and Scaff Haddad Bartos ([12], Brazil), analyzed how organizational or bureaucratic systems can influence the degree of vulnerability that populations in hospitals or prisons can have. A common denominator in these editorials is the lack of health services available in these settings, where mental health is notably absent. Unless existing normative frameworks (e.g., those in Brazilian prisons [12]) that compel the inclusion of mental health professionals in primary care teams are strengthened, the mental health gaps between the most and the less vulnerable population groups in Latin America will continue to widen.

Lastly, authors emphasized the need to address the social determinants of health for reducing health inequalities and preventing the structuring of noxious social contexts and their harmful health consequences. The editorial by Werneck et al. ([13], Brazil) and by Sánchez ([14], Mexico) explained clearly why health promotion initiatives on physical activity in Brazil or on drug abuse in Mexico are bound to fail unless the broader “causes of the causes” of health and illness are considered when designing public health interventions. As long as the most vulnerable populations are deprived of decent jobs, denied basic urban development, and entangled in gender inequalities, any public health effort aimed at promoting healthy lifestyles, or preventing interpersonal violence, will be halted by the harsh reality of structural conditions that determine the success or failure of public health policy.

We conclude from the YREs of this call, that the most pressing public health challenges in Latin America are not isolated issues but rather symptoms of deeper underlying conditions. The inequalities, spanning from economic, gender, and social dimensions, are highlighted by young researchers in the region as the drivers of adverse public health outcomes, impacting the most vulnerable populations. Reaching out to these populations by strengthening health systems and designing intersectoral policies that address the social determinants of health seems to be the best strategies for ending health inequality in the region.
